# Neoadjuvant chemoradiotherapy versus neoadjuvant chemotherapy alone for patients with locally advanced rectal cancer: a propensity-score-matched analysis combined with SEER validation

**DOI:** 10.1007/s00432-023-04779-y

**Published:** 2023-05-08

**Authors:** Jingjing Wu, Mingzhe Huang, Yuanhui Wu, Yisong Hong, Linbin Cai, Rongzhao He, Yanxin Luo, Puning Wang, Meijin Huang, Jinxin Lin

**Affiliations:** 1 Department of Colorectal Surgery, Department of General Surgery, The Sixth Affiliated Hospital, Sun Yat-Sen University, Guangzhou, Guangdong People’s Republic of China; 2 Guangdong Provincial Key Laboratory of Colorectal and Pelvic Floor Diseases, The Sixth Affiliated Hospital, Sun Yat-Sen University, Guangzhou, Guangdong People’s Republic of China; 3 Guangdong Institute of Gastroenterology, Guangzhou, Guangdong People’s Republic of China

**Keywords:** Neoadjuvant chemoradiotherapy, Neoadjuvant chemotherapy, Survival, Postoperative complications, Propensity-score-matched analysis, SEER

## Abstract

**Background:**

Neoadjuvant therapy followed by radical surgery is recommended for locally advanced rectal cancer (LARC). But radiotherapy can cause potential adverse effects. The therapeutic outcomes, postoperative survival and relapse rates between neoadjuvant chemotherapy (N-CT) and neoadjuvant chemoradiotherapy (N-CRT) patients have rarely been studied.

**Methods:**

From February 2012 to April 2015, patients with LARC who underwent N-CT or N-CRT followed by radical surgery at our center were included. Pathologic response, surgical outcomes, postoperative complications and survival outcomes (including overall survival [OS], disease-free survival [DFS], cancer-specific survival [CSS] and locoregional recurrence-free survival [LRFS]) were analyzed and compared. Concurrently, the Surveillance, Epidemiology, and End Results Program (SEER) database was used to compare OS in an external source.

**Results:**

A total of 256 patients were input into the propensity score-matching (PSM) analysis, and 104 pairs remained after PSM. After PSM, the baseline data were well matched and there was a significantly lower tumor regression grade (TRG) (*P* < 0.001), more postoperative complications (*P* = 0.009) (especially anastomotic fistula, *P* = 0.003) and a longer median hospital stay (*P* = 0.049) in the N-CRT group than in the N-CT group. No significant difference was observed in OS (*P* = 0.737), DFS (*P* = 0.580), CSS (*P* = 0.920) or LRFS (*P* = 0.086) between the N-CRT group and the N-CT group. In the SEER database, patients who received N-CT had similar OS in both TNM II (*P* = 0.315) and TNM III stages (*P* = 0.090) as those who received N-CRT.

**Conclusion:**

N-CT conferred similar survival benefits but caused fewer complications than N-CRT. Thus, it could be an alternative treatment of LARC.

**Supplementary Information:**

The online version contains supplementary material available at 10.1007/s00432-023-04779-y.

## Introduction

Preoperative chemoradiotherapy combined with radical resection followed by total mesorectal excision (TME) is recommended as standard treatment for patients with locally advanced rectal cancer (LARC) (Sauer et al. 2004; Benson et al. 2020). After preoperative neoadjuvant radiotherapy (N-RT) combined with TME, a lower rate of local recurrence was observed for local recurrence, but survival benefits were not found (Marijnen et al. 2002; Peeters et al. 2007; Tiefenbacher and Wenz 2001; Gijn et al. 2011). Furthermore, N-RT has been reported to raise potential safety concerns, such as increased rates of bowel dysfunction, anal mucous loss, anal blood loss, incontinence and sexual dysfunction (Peeters et al. 2005; Lange et al. 2007; Bruheim et al. 2010). Thus, the clinical benefit and adverse effects of N-RT should be carefully weighed.

To date, only a few studies have focused on the benefits of neoadjuvant chemotherapy (N-CT) compared with neoadjuvant chemoradiotherapy (N-CRT) for LARC patients. In the present study, we conducted 1:1 propensity-score-matching (PSM) analysis to compare the therapeutic response, surgical outcomes, and prognosis of patients who received N-CT versus N-CRT followed by TME at our center. Then, we explored the Surveillance, Epidemiology, and End Results Program (SEER) database to compare OS in this external source.

## Patients and methods

### Study design and patient enrollment

**Fig. 1:**
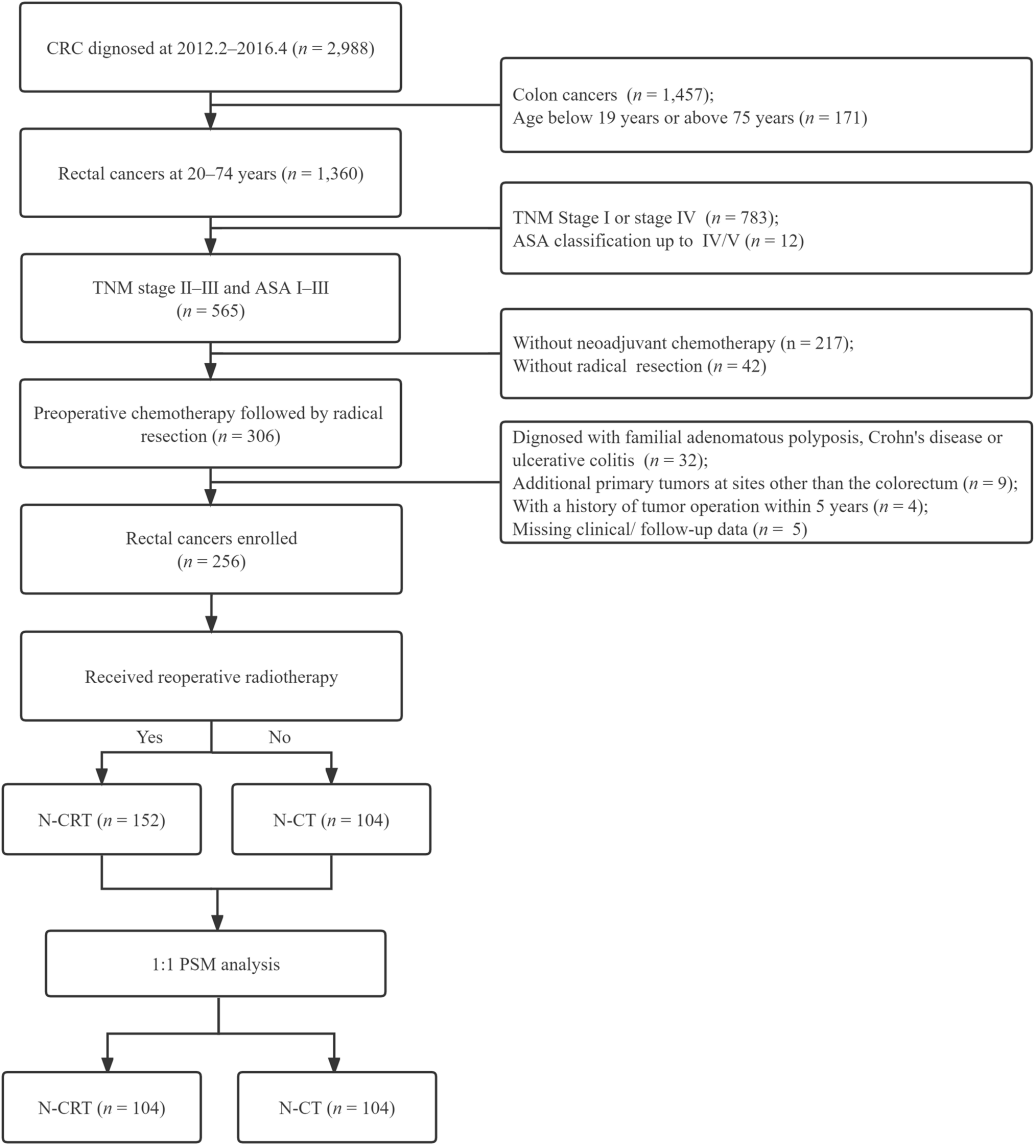
Study flowchart of the center

Among 1531 hospitalized patients with rectal cancer (RC) between February 2012 and April 2015, 256 patients were recruited for this retrospective nonrandomized study, and their data were collected from the clinical database of colorectal cancer (CRC) at the Sixth Affiliated Hospital, Sun Yat-sen University (Guangzhou, China). The inclusion criteria were as follows: (1) an initial diagnosis of stage T3–4 and/or N+ disease in the TNM classification according to the American Joint Committee on Cancer-International Union Against Cancer (7th edition); (2) aged from 20 to 74 years and either sex; (3) American Society of Anesthesiologists (ASA) physical status scores of 1–3 points; (4) rectal adenocarcinoma confirmed by preoperative biopsy and/or postoperative pathology; and (5) N-CT or N-CRT followed by radical surgery (R0 resection). The exclusion criteria were as follows: (1) familial adenomatous polyposis, Crohn’s disease or ulcerative colitis; (2) metastatic or recurrent cancer; (3) additional primary tumors at sites other than the colorectum; (4) a history of tumor surgery within 5 years; or (5) missing clinical/follow-up data. The detailed participant selection process is shown in the flowchart (Fig. 1).

### Treatment

Radiotherapy was delivered at a dose of 1.8–2.0 Gy daily on weekdays for a total of 23–28 fractions over 5–6 weeks and a total dose of 44.0–50.0 Gy unless they were intolerant. Radiation was delivered with a minimum energy of 6-MV photons through a three-field or four-field box technique to the primary tumor and to mesorectal, presacral, and internal iliac LNs. All radiation oncologists received standard training in radiation treatment.

Preoperative N-CT included fluorouracil and mFOLFOX6 regimens. The treatment modes were 5-fluorouracil-radiotherapy (N-CRT), FOLFOX6-radiotherapy (N-CRT), mFOLFOX6 (N-CT) and 5-fluorouracil (N-CT). Patients who received preoperative fluorouracil-radiotherapy treatment accepted five cycles of infusional fluorouracil (leucovorin 400 mg/m^2^ intravenously drip, then fluorouracil 400 mg/m^2^ intravenously drip, fluorouracil 2.4 g/m^2^ by 48-h continuous intravenous infusion [de Gramont regimen]) with concurrent radiotherapy during cycles 2–4 and postoperative adjuvant chemotherapy with seven cycles of fluorouracil. Patients who underwent mFOLFOX6-radiotherapy received the same intravenous drugs as the fluorouracil-radiotherapy group plus oxaliplatin 85 mg/m^2^ administered intravenously on day 1 of each cycle. These patients received mFOLFOX6 treatment as N-CT for 4–6 cycles and 6–8 cycles postoperatively, respectively.

To obtain minimum local recurrence rates, surgical complete tumor resection is the key factor and central goal of high-quality RC operation. Mesorectal excision was carried out by the laparoscopic or open method as described (Heald et al. 1982). The rectum was dissociated and the attached mesorectum with more than a 4 cm distal clearance margin to the tumor was completely resected. Anterior resection or abdominoperineal resection was selected based on the tumor location, Eastern Cooperative Oncology Group performance status, and the wishes of the patient and his or her family members.

### Study measurements

Tumors were clinically classified into stage II/III by magnetic resonance imaging (MRI) and/or computed tomography (CT) plus endorectal ultrasound. A positive node was defined as having a diameter ≥ 1.0 cm on imaging. Fiberoptic colonoscopy with pathological biopsy was carried out regularly. Routine digital rectal examination was implemented. The most distal border was located 12 cm from the anal verge. During the study, patients received pelvic cavity MRI and abdominal plus pelvic CT within 2 weeks before initiation of N-CT, once every two periods of chemotherapy, and within 2 weeks before radical resection. The definition and classification of anastomotic fistula (AF) were based on the International Study Group of Rectal Cancer definitions and classification system (Rahbari et al. 2010). Grade A AF was not accompanied by clinical symptoms or abnormal laboratory tests. Generally, no active therapeutic intervention was required for this mild form. AF was designated as grade B when the patient’s clinical profile required an active therapeutic intervention without reoperation. Increased laboratory indicators of infection and purulent/fecal drain contents could be observed in these patients. Grade B AF can be commonly managed by the administration of antibiotics and/or radiologic placement of a pelvic drain or transanal lavage. Patients with grade C AF usually have an unstable condition and certain symptoms (abdominal pain, fever), and they may subsequently develop signs of peritonitis and require relaparotomy. Early AF was defined as leakage diagnosed within 30 days, and late AF was defined as leakage diagnosed after 30 days postoperatively.

After neoadjuvant therapy, pathological complete response (pCR) was defined as the absence of viable tumor cells in the primary tumor and LNs (ypT0N0) as evaluated by two pathologists blinded to the treatment and outcome. ypTCR referred to the absence of a primary tumor by postoperative pathology regardless of whether LNs were positive or negative. ypNCR was defined as no positive LNs detected by histologic examination without considering the primary rectal tumor, while positive LNs were first definitely diagnosed by imaging examination. The pathological tumor regression grading (TRG) system, with four-point grading, was classified according to the 2010 American Joint Committee on Cancer system modification from the Ryan TRG system based on the volume of residual primary tumor cells (Ryan et al. 2005; Kim et al. 2016).

For the first 3 years after surgery, patients were followed-up every 6 months and then once a year during subsequent years. Regular assessments included physical examination, laboratory tests, fiberoptic colonoscopy, and imaging. OS, disease-free survival (DFS), cancer-specific survival (CSS) and locoregional recurrence-free survival (LRFS) were recorded and analyzed.

### SEER database

We identified 8194 patients with diagnosed LARC who underwent chemoradiotherapy from the 18 population-based SEER registries (with additional treatment fields) between 2012 and 2015. Patients were selected according to the following criteria: (1) age 20–74 years; (2) lesions located in the rectum; (3) pathological diagnosis of malignant tumors; (4) adenocarcinoma; (5) TNM stage II–III disease; (6) both surgery and chemotherapy; and (7) no missing survival data. The selection process including the inclusion and exclusion criteria is shown in Fig. S1.

### Statistical analysis

Statistical analysis was carried out using SPSS version 26.0 (SPSS Inc., Chicago, IL, USA) and R software (The R Foundation for Statistical Computing, Vienna, Austria; https://www.Rproject.org) for Windows. Figures were made in Prism version 8.0 (GraphPad Software, San Diego, California, USA). A two-sided P value less than 0.05 was considered statistically significant. According to the distribution characteristics, continuous variables are presented as the mean ± standard deviation (SD) or median (quartiles), and categorical data are presented as the number (*n*) and proportion (%). Continuous variables were compared between groups by Student’s *t* test when they followed a normal distribution; otherwise, the Mann–Whitney *U* nonparametric test was applied. Comparisons of categorical variables were made using the *χ*^2^ test or Fisher’s exact test. Survival was analyzed with the Kaplan–Meier method and was assessed by the log-rank test. Binary logistic regression was carried out to explore the risk factors for AF.

PSM was performed to adjust for differences in the baseline data as described by Rubin and Rosenbaum (Rosenbaum and Rubin 1983). A caliper width of 0.4 was used to assess both preferable homogeneity and a minor decrease in sample size. The parameters included in the model can be seen in Table S1.

## Results

### Demographics and clinicopathologic features of all LARC patients

A total of 256 eligible patients diagnosed with LARC who received N-CT or N-CRT followed by radical resection of RC at our center were included (Table S1). The median follow-up time was 69.8 months. All patients (179 males and 77 females) had a mean age of 53.2 ± 12.1 years. Among them, the majority of patients (*n* = 140, 54.7%) had a distance from the lesion to the anus of 5–10 cm, and only a very small percentage of patients (5.5%, *n* = 14) had tumors over 10 cm. Specifically, there were 152 and 104 patients in the N-CRT and N-CT groups, respectively. Most of the baseline indicators, including clinical T stage and N stage, were not significantly different between the two groups. However, patients who underwent N-CRT had lower RC (*P* = 0.020) and a later clinical TNM stage (*P* = 0.014) than those who received N-CT.

In the SEER set, 6386 patients underwent preoperative chemoradiotherapy. Most of the patients (*n* = 6129) received N-CRT, and only 257 were treated with N-CT (Fig. S1).

### Comparison of clinicopathologic characteristics after PSM

After 1:1 PSM, 208 patients were matched, which included 104 patients who underwent N-CRT and 104 patients who underwent N-CT treatment. All demographic data (including age, sex and body mass index), laboratory indexes (such as routine blood tests, coagulation function, liver function, renal function, CEA), and tumor parameters (including maximum diameter, distance from anus, clinical T stage, clinical N stage, and clinical TNM stage) were well matched (Table 1).

**Table 1:** Baseline data between the patients who received neoadjuvant chemoradiotherapy (CRT) and those who received neoadjuvant chemotherapy (CT) after propensity-score matching (PSM)

Characteristic	All patients (*n* = 208)	Patients with N-CRT (*n* = 104)	Patients with N-CT (*n* = 104)	*P* value
Age, years, mean ± SD	53.2 ± 12.2	52.9 ± 11.4	53.6 ± 12.9	0.641^a^
Male/female, *n* (%)	147 (70.7)/61 (29.3)	73 (70.2)/31 (29.8)	74 (71.2)/30 (28.8)	0.879^b^
BMI, kg/m^2^, mean ± SD	22.9 ± 3.5	23.0 ± 3.2	22.8 ± 3.9	0.630^a^
Albumin, g/L, mean ± SD	43.3 ± 3.8	43.5 ± 3.4	43.1 ± 4.2	0.449^a^
CEA, U/mL, median (quartile)	2.7 (1.5–6.1)	2.6 (1.4–6.0)	2.9 (1.8–6.1)	0.399^c^
Tumor diameter, cm, mean ± SD	4.3 ± 1.5	4.3 ± 1.5	4.3 ± 1.6	0.839^a^
*Distance from anus, cm*				
< 5	78 (37.5)	43 (41.3)	35 (33.7)	0.302^c^
5–10	118 (56.7)	55 (52.9)	63 (60.6)	
> 10	12 (5.8)	6 (5.8)	6 (5.8)	
Mean distance, cm, mean ± SD	6.1 ± 2.6	5.8 ± 2.6	6.4 ± 2.6	0.091^a^
*Clinical T stage, n (%)*				
cT4b	14 (6.7)	9 (8.7)	5 (4.8)	0.940^c^
cT4a	10 (4.8)	4 (3.8)	6 (5.8)	
cT3	179 (86.1)	87 (83.7)	92 (88.5)	
cT2	5 (2.4)	4 (3.8)	1 (1.0)	
*Clinical N stage, n (%)*				
cN2b	38 (18.3)	24 (23.1)	14 (13.5)	0.510^c^
cN2a	33 (15.9)	13 (12.5)	20 (19.2)	
cN1	91 (43.8)	44 (42.3)	47 (45.2)	
cN0	46 (22.1)	23 (22.1)	23 (22.1)	
*Clinical TNM stage, n (%)*				
IIA	42 (20.2)	20 (19.2)	22 (21.2)	0.352^c^
IIB/IIC	4 (1.9)	3 (2.9)	1 (1.0)	
IIIA	5 (2.4)	4 (3.8)	1 (1.0)	
IIIB	112 (53.8)	49 (47.1)	63 (60.6)	
IIIC	45 (21.6)	28 (26.9)	17 (16.3)	

*BMI* body mass index, *CEA* carcinoembryonic antigen

^a^Student’s *t* test

^b^Pearson’s χ^2^

^c^Wilcoxon rank sum test

Although there was a tendency in the SEER database toward a higher proportion of patients with stage T4b disease and a lower rate of patients with T3 disease among those who underwent CT alone (*P* = 0.074), no significant difference in the baseline data was observed between patients who underwent N-CRT and N-CT (Table 2). Thus, PSM analysis was unnecessary.

**Table 2:** Baseline data of the patients who received neoadjuvant chemoradiotherapy (N-CRT) and neoadjuvant chemotherapy (N-CT) in the SEER database

Characteristics	Patients with N-CRT (*n* = 6129)	Patients with N-CT (*n* = 257)	*P* value
Age, years, mean ± SD	56.8 ± 10.1	56.5 ± 10.8	0.629^a^
Male/female, *n* (%)	3816 (62.3)/2313 (37.7)	168 (65.4)/89 (34.6)	0.314^b^
*CEA*			
Positive	1957 (31.9)	88 (34.2)	0.941^c^
Borderline	24 (0.4)	0 (0.0)	
Negative	2419 (39.5)	99 (38.5)	
Unknown	1729 (28.2)	70 (27.2)	
*Tumor diameter, cm, n (%)*			
< 3	964 (15.7)	47 (18.3)	0.202^c^
3–5	1754 (28.6)	82 (31.9)	
5–7	1708 (27.9)	57 (22.2)	
> 7	743 (12.1)	27 (10.5)	
Unknown	960 (15.7)	44 (17.1)	
*Race*			
White	4969 (81.1)	205 (79.8)	0.526^c^
American Indian/Alaska Native	53 (0.9)	4 (1.6)	
Black	540 (8.8)	18 (7.0)	
Asian	542 (8.8)	28 (10.9)	
Unknown	25 (0.4)	2 (0.8)	
*Degree of differentiation*			
Well	414 (6.8)	17 (6.6)	0.201^c^
Moderate	4267 (69.6)	189 (73.5)	
Poor	515 (8.4)	23 (8.9)	
Undifferentiated/anaplastic	65 (1.1)	1 (0.4)	
Unknown	868 (14.2)	27 (10.5)	
*Year of diagnosis*			
2012	1422 (23.2)	53 (20.6)	0.130^c^
2013	1465 (23.9)	56 (21.8)	
2014	1681 (27.4)	74 (28.8)	
2015	1561 (25.5)	74 (28.8)	
*Pathological type*			
Adenocarcinoma	5858 (95.6)	244 (94.9)	0.628^b^
Mucinous adenocarcinoma	271 (4.4)	13 (5.1)	
*Perineural invasion*			
Positive	645 (10.5)	32 (12.5)	
Negative	4663 (76.1)	184 (71.6)	
Unknown	821 (13.4)	32 (12.5)	
*T stage, n (%)*			
T4b	497 (8.1)	32 (12.5)	0.074^c^
T4a	166 (2.7)	6 (2.3)	
T4NOS	26 (0.4)	1 (0.4)	
T3	4966 (81.0)	202 (78.6)	
T2	345 (5.6)	12 (4.7)	
T0-1	85 (1.4)	3 (1.2)	
T0	4 (0.1)	0 (0.0)	
Tx	44 (0.7)	1 (0.4)	
*N stage, n (%)*			
N2b	246 (4.0)	13 (5.1)	0.696^c^
N2a	426 (7.0)	12 (4.7)	
N2NOS	152 (2.5)	4 (1.6)	
N1c	111 (1.8)	5 (1.9)	
N1b	769 (12.5)	38 (14.8)	
N1a	958 (15.6)	36 (14.0)	
N1NOS	1215 (19.8)	52 (20.2)	
N0	2252 (36.7)	97 (37.7)	
*cTNM stage, n (%)*			
IIA	2006 (32.7)	80 (31.1)	0.673^c^
IIB	65 (1.1)	2 (0.8)	
IIC	171 (2.8)	14 (5.4)	
IINOS	10 (0.2)	1 (0.4)	
IIIA	386 (6.3)	13 (5.1)	
IIIB	2787 (45.5)	113 (44.0)	
IIIC	552 (9.0)	31 (12.1)	
IIINOS	152 (2.5)	3 (1.2)	

*CEA* carcinoembryonic antigen

^a^Student’s *t* test

^b^Pearson’s *χ*^2^

^c^Wilcoxon rank sum test

### Postsurgical pathologic response after PSM

Before treatment, both the pathological type (*P* = 0.773) and degree of differentiation (*P* = 0.489) by biopsy were comparable between the two groups (Table 3). There was no difference in regimen (*P* = 0.479) and the median number of chemotherapy cycles before and after operation were 5, and 7, respectively. The rate of ypCR significantly increased after radiotherapy (*P* = 0.004). Specifically, the rate of ypTCR was 27.9% (*n* = 29) versus 8.7% (*n* = 9) (*P* = 0.011), and the rate of ypNCR was 59.6% (*n* = 62) versus 52.9% (*n* = 55) (*P* = 0.328) between the N-CRT and the N-CT groups, respectively. These results indicated that radiotherapy only resulted a decrease in T stage (*P* = 0.003) but not N stage (*P* = 0.239). Except for one patient with treatment intolerance who underwent only 2 cycles of N-CT, almost all patients (99.6%) completed the 90% N-CT plan, and a total of 100 patients (96.2%) completed the 90% N-CRT plan. Likewise, the two groups differed significantly in terms of TRG (*P* < 0.001), and the N-CRT group had a higher pathological tumor remission rate. Additional details are shown in Table 3.

**Table 3:** Details of N-CRT and N-CT and pathologic outcome of LARC patients after treatment

Variables	Patients with CRT (*n* = 104)	Patients with CT (*n* = 104)	*P* value
*Pathological types by biopsy*^*#*^			
Adenocarcinoma	98 (94.2)	99 (95.2)	0.773^a^
Mucinous adenocarcinoma	5 (4.8)	3 (2.9)	
Unknown	1 (1.0)	2 (1.9)	
*Degree of differentiation by biopsy*^*#*^			
Well differentiated	38 (36.5)	43 (41.3)	0.489^a^
Moderately differentiated	50 (48.1)	47 (45.2)	
Poor differentiated	13 (12.5)	10 (9.6)	
Unknown^¶^	3 (2.9)	4 (3.8)	
Vessel invasion positive	3 (2.9)	6 (5.8)	0.496^b^
Perineural invasion positive	4 (3.8)	2 (1.9)	0.678^b^
Mesorectal fascia positive	28 (27.5)	25 (24.5)	0.632^c^
ypCR	24 (23.1)	9 (8.7)	**0.004**^c^
ypNCR	62 (59.6)	55 (52.9)	0.328^c^
> 90% chemotherapy plan completed	104 (100.0)	103 (99.0)	1.000^b^
> 90% Radiotherapy plan completed	100 (96.2)	–	–
Radiotherapy dosage	50 (48.1)	–	–
*ypT downstaging*^*¶*^			
Progression	0 (0.0)	1 (1.0)	**0.003**^a^
0	37 (35.6)	50 (48.1)	
1	26 (25.0)	32 (30.8)	
2	14 (13.5)	12 (11.5)	
3	25 (24.0)	7 (6.7)	
5	2 (1.9)	2 (1.9)	
*ypN downstaging*^*¶*^			
Progression	3 (2.9)	7 (6.7)	0.239^a^
0	30 (28.8)	32 (30.8)	
1	40 (38.5)	40 (38.5)	
2	15 (14.4)	12 (11.5)	
3	16 (15.4)	13 (12.5)	
*ypTNM downstaging*^¶^			
Progression	3 (2.9)	7 (6.7)	**0.046**^a^
0–1	30 (28.8)	39 (37.5)	
2–3	9 (8.7)	2 (1.9)	
4–5	40 (38.5)	46 (44.2)	
≥ 6	22 (21.2)	10 (9.6)	
*ypTNM stage*			
0	24 (23.1)	9 (8.7)	0.060^a^
I	24 (23.1)	34 (32.7)	
II	35 (33.7)	30 (28.8)	
III	21 (20.2)	31 (29.8)	
*TRG*			
0	29 (27.9)	9 (8.7)	**< 0.001**^**a**^
1	39 (37.5)	23 (22.1)	
2	35 (33.7)	46 (44.2)	
3	1 (1.0)	26 (25.0)	
*Pathological type*			
ypTCR	29 (27.9)	9 (8.7)	**0.011**^a^
Adenocarcinoma	66 (63.5)	89 (85.6)	
Mucinous adenocarcinoma	9 (8.7)	6 (5.8)	
*Degree of differentiation*^*#*^			
ypTCR	29 (27.9)	9 (8.7)	0.086^a^
Well differentiated	24 (23.1)	37 (35.6)	
Moderately differentiated	35 (33.7)	43 (41.3)	
Poor differentiated	13 (12.5)	12 (11.5)	
Unknown^¶^	3 (2.9)	3 (2.9)	

*TRG* tumor regression grading

^a^Wilcoxon rank sum test

^b^*χ*^2^ with Yates’ correction

^c^Pearson’s *χ*^2^

^¶^According to the classification of T stage, there are six T stages: T0, T1, T2, T3, T4a, and T4b. For N stage, there are four N stages: N0, N1, N2a, and N2b. There are eight T stages: 0, I, IIA, IIB, IIC, IIIA, IIIB, and IIIC

^#^Owing to the limitations of biopsy sampling, some pathological types and degrees of differentiation were not obtained before treatment in a few cases. Postoperative pathological specimens were indiscernible after neoadjuvant therapy on rare occasions

### Surgical outcomes and postoperative complications

The overall sphincter preservation rate of all LARC patients who received radical resection was 90.2%. More specifically, anal preservation was achieved in 133 (87.5%) and 98 (94.2%) patients in the N-CRT group and the N-CT group, respectively. Most patients (*n* = 235, 91.8%) underwent laparoscopic surgery. Patients treated with N-CRT exhibited a higher incidence of complications than those treated with N-CT (43.4% versus 30.8%, *P* = 0.025). Patients diagnosed with early AF in the N-CRT group had a slightly higher incidence (*P* = 0.153) (*n* = 10, 6.6%) than those in the N-CT group (*n* = 2, 1.9%). As follow-up continued, the difference in the rate of AF became significant between the two groups (20.4% versus 8.7%, *P* = 0.011). Additional details are presented in Table S2.

After PSM, similar results were observed between the two groups. Patients in the N-CRT group also suffered from higher percentages of postoperative complications (*n* = 50, 48.1% versus *n* = 32, 30.7%, *P* = 0.009) and longer postoperative hospital stays (median 12 days, IQR 9–16 days versus median 11 days, IQR 9–14 days, *P* = 0.049) than the N-CT group. More significantly, patients who received preoperative radiotherapy displayed a higher rate of AF not only in the short term (*P* = 0.030) but also in the long term (*P* = 0.003). No 30-day mortality was observed. The surgical operation and complication data are shown in Table 4.

**Table 4:** Surgical anesthesia outcomes after PSM

Variables	All patients (*n* = 208)	Patients with CRT (*n* = 104)	Patients with CT (*n* = 104)	*P* value
Preoperative intestinal obstruction	4 (1.9)	3 (2.9)	1 (1.0)	0.614^a^
*ASA (%), n (%)*				
I	32 (15.4)	18 (17.3)	14 (13.5)	0.454^b^
II	161 (77.4)	79 (76.0)	82 (78.8)	
III	15 (7.2)	7 (6.7)	8 (7.7)	
Anal preservation	189 (90.9)	91 (87.5)	98 (94.2)	0.092^c^
*Surgical procedure*				
Open	15 (7.2)	6 (5.8)	9 (8.7)	0.292^b^
Laparoscope	192 (92.3)	98 (94.2)	94 (90.4)	
Trans-anal resection	1 (0.5)	0 (0.0)	1 (1.0)	
Operation duration	241.3 ± 78.1	246.3 ± 73.7	236.2 ± 82.2	0.351^d^
*Morbidity, n (%)*				
NO	126 (60.6)	54 (51.9)	72 (69.2)	**0.009**^b^
Clavien grade I–II	70 (33.7)	40 (38.5)	30 (28.8)	
Clavien grade III–IV	12 (5.8)	10 (9.6)	2 (1.9)	
AF within 30 days	11 (5.3)	9 (8.7)	2 (1.9)	**0.030**^c^
AF in the whole course	34 (16.3)	25 (24.0)	9 (8.7)	**0.003**^c^
Urinary complications	11 (5.3)	6 (5.8)	5 (4.8)	0.757^c^
Blood transfusion	10 (4.8)	8 (7.7)	1 (1.0)	0.052^c^
Reoperation	3 (1.4)	3 (2.9)	0 (0)	0.245^a^
Postoperative hospital stays	11 (9–15)	12 (9–16)	11 (9–14)	**0.049**^b^

*ASA* American Society of Anesthesiologists, *AF* anastomotic fistula

^a^*χ*^2^ with Yates’ correction

^b^Wilcoxon rank sum test

^c^Pearson’s χ^2^

^d^Student's *t* test

### Classification and risk factors for AF

For further investigation, we analyzed the grades of AF. There were 34 patients with AF and 174 patients without AF. Interestingly, only half of the cases of AF occurred within 90 days after surgery, and the proportion of early AF cases was less than 1/3 of the total. The postoperative rates of grade A, grade B, and grade C AF were 35.3%, 50.0%, and 14.7%, respectively (Table S3). Three patients underwent surgical treatment in both the early AF (27.3%) and late AF (13.0%) groups (*P* = 0.087). Patients who presented with AF suffered from a longer median hospital stay than those without AF (13.0 versus 11.0, *P* = 0.014) (not listed in the table).

Binary logistic regression was carried out to explore the risk factors for AF. The initial indexes included can be found in Tables S4 and S5. After univariate analysis, two factors, sex and radiotherapy, were ultimately included in the multivariate analysis. Radiotherapy was identified as an independent risk factor for AF both before PSM (HR: 3.192, 95% CI: 1.394–7.313, *P* = 0.006) and after PSM (HR: 3.455, 95% CI: 1.511–7.898, *P* = 0.003, Table S6).

### Survival analysis

**Fig. 2:**
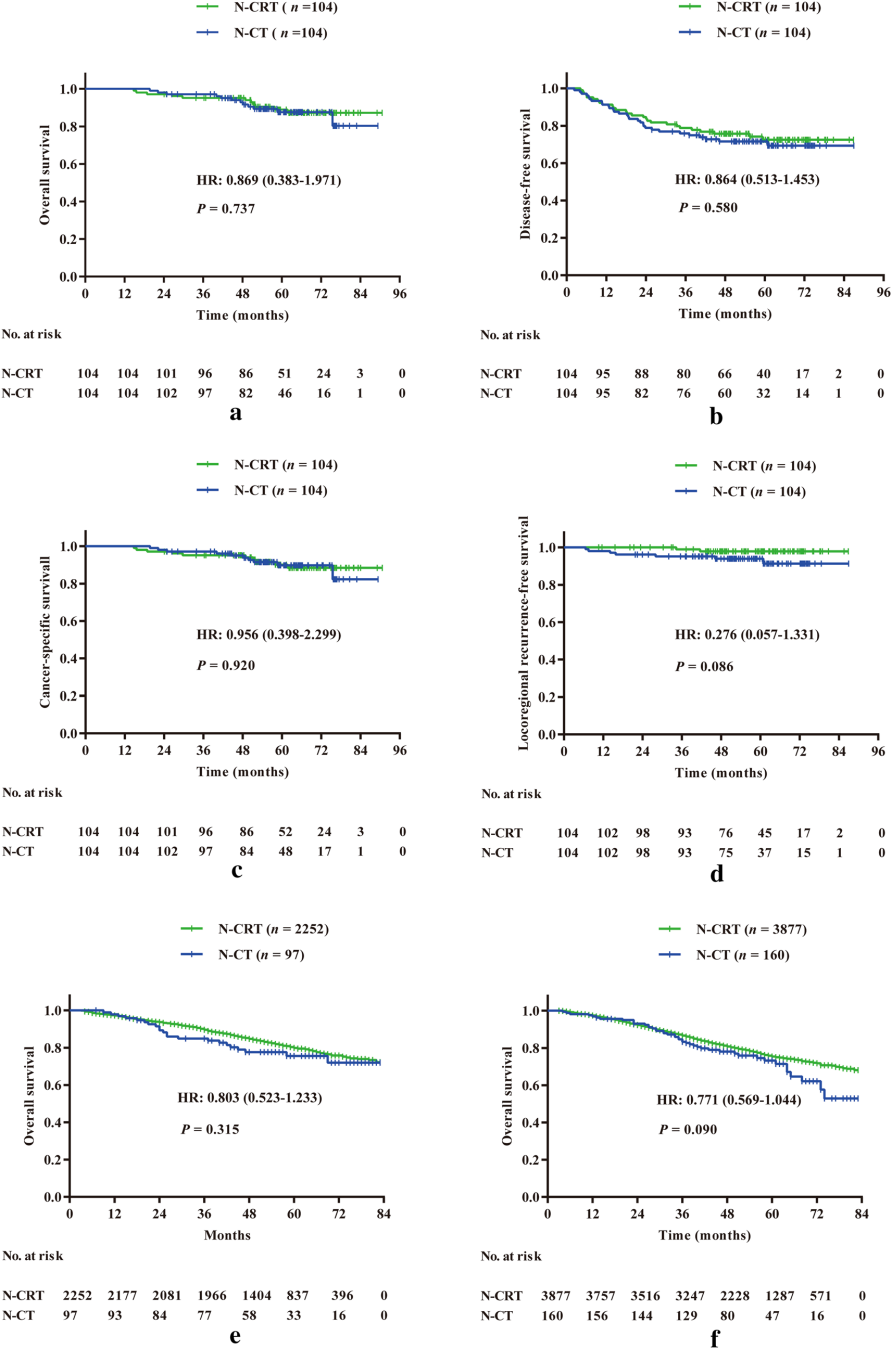
Survival analysis of the N-CRT and N-CT groups. No significance of overall survival (**a**), disease-free survival (**b**), cancer-specific survival (**c**), and locoregional recurrence-free survival (**d**) was observed after propensity-score-matching analysis at our center. Similarly, there was no significance comparing the overall survival analysis of stage II (**e**) and stage III (**f**) LARC patients in the N-CRT and N-CT groups in the SEER database

After PSM, the median OS was more than 59.0 months for the 208 patients. Fifty-seven patients were recorded with recurrent or progressive disease during the period of follow-up. The recurrence/progression rate was 28.4% for the N-CT group versus 25.8% for the N-CRT group. No significant differences were found between the N-CRT and N-CT groups in OS (*P* = 0.737) (Fig. 2a), DFS (*P* = 0.580) (Fig. 2b) or CSS (*P* = 0.920) (Fig. 2c). Although there seemed to be a trend of better LRFS for the N-CRT group than for the N-CT group, radiotherapy did not provide significantly better LRFS than chemotherapy alone (*P* = 0.086) (Fig. 2d). Furthermore, we analyzed survival in the TNM stage II and III subgroups. No significant difference between N-CRT and N-CT was found in the OS, DFS, CSS or LRFS of stage II (Fig. S2a–d) or stage III patients (Fig. S3a–d).

In the SEER database, borderline significance for OS was observed (*P* = 0.050) (Fig. S4). Given the tendency of a higher proportion of T4b stage in the N-CT group (*P* = 0.074), Cox regression analysis was carried out to adjust the variable. After adjustment, no significant difference (*P* = 0.089; HR, 1.113; 95% CI, 0.984–1.260) was found between the N-CRT and the N-CT groups (data not shown). More importantly, no significant difference in OS between the N-CRT and N-CT groups was found in either the stage II (*P* = 0.315) (Fig. 2e) or stage III patient subgroups (*P* = 0.090) (Fig. 2f).

**Fig. 3:**
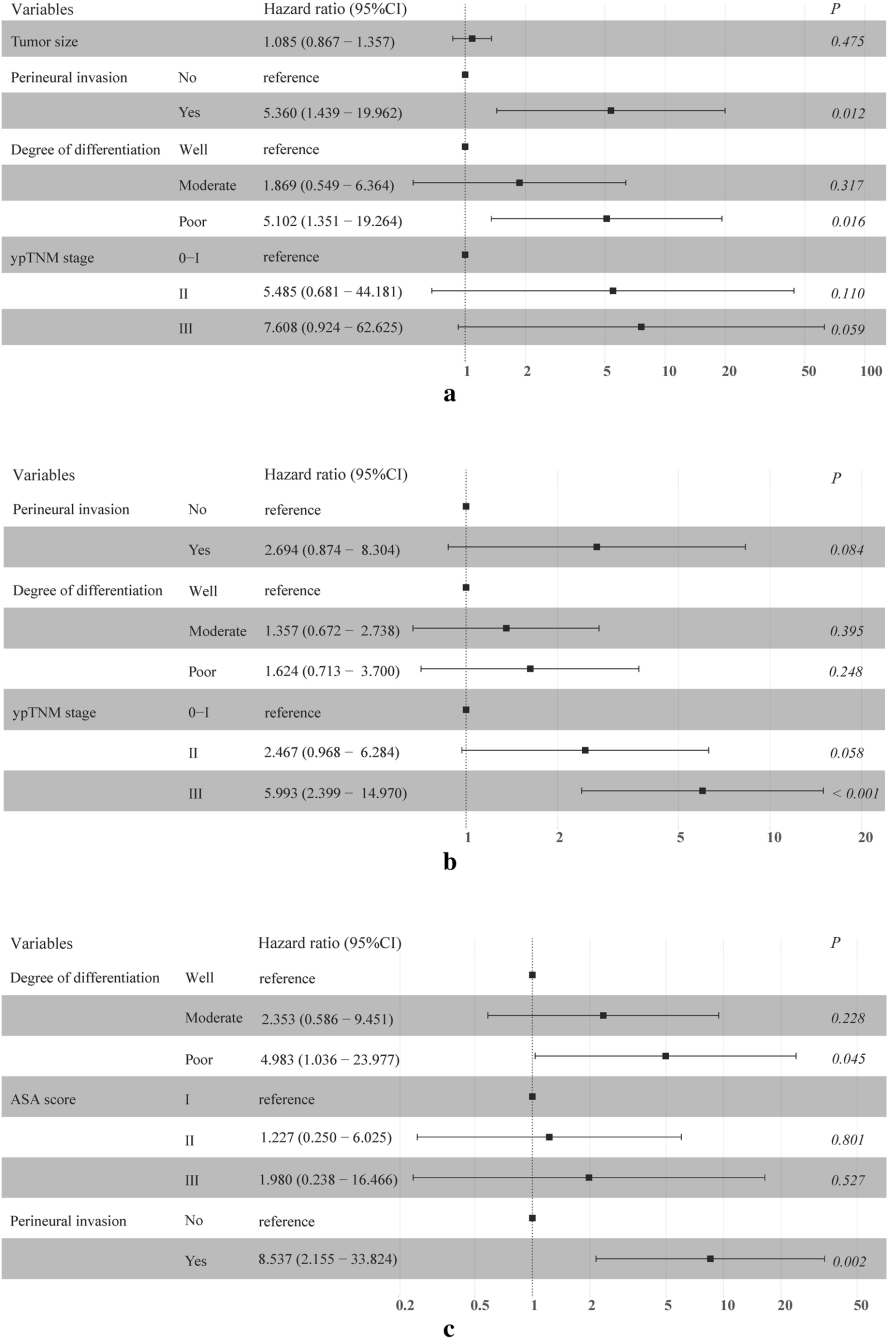
Forest plot of the risk factors confirmed by multivariate analysis influencing survival: overall survival (**a**), disease-free survival (**b**) and locoregional recurrence-free survival (**c**) at our center

A forest plot was generated to detect the independent risk factors for OS (Fig. 3a), DFS (Fig. 3b) and LRFS (Fig. 3c). Variables with *P* values less than 0.05 in the univariate analysis were included in the forest plot. A late ypTNM stage was identified as an independent risk factor for OS and DFS. In addition, perineural invasion and poor pathological differentiation were independent risk factors for unfavorable OS.

## Discussion

Routine N-CT and some patients received radiotherapy is the standard treatment for LARC recommended by the NCCN and EMSO guidelines (Benson et al. 2020; Glynne-Jones et al. 2018). In this investigation, we found that preoperative chemotherapy alone can yield similar benefits for OS as chemoradiotherapy in both our center and the SEER database. Radiotherapy cannot prolong not only the OS time but also the DFS and CSS of LARC patients. In addition, there is no denying that radiotherapy can substantially increase the risk of AF, and this side effect might be a long-term outcome after surgery.

After it was first proposed by Heald and Ryall in 1986, TME was perceived as the gold standard operation modality for RC (Enker et al. 1995; Heald and Ryall 1986). Through the 1990s, a few studies introduced training workshops of TME and emphasized the idea of “specimen-orientated surgery”, and this surgical procedure was reported to reduce the postoperative local recurrence rates for RC from more than 15% to approximately 5% at the population level. Previous studies have also suggested that after stratification analysis, the local recurrence rate after TME is still as high as 24.1%, with the distant metastasis rate being 26–36% for middle/low RC after R0 resection or for stage III RC (Gijn et al. 2011; Enker et al. 1999; Bonadeo et al. 2001; Akagi et al. 2013). For LARC, given the high risk for disease recurrence/metastasis, surgical treatment alone is unable to yield optimal outcomes (Douglass et al. 1986). Radiotherapy uses high-energy radiation to exert is a killing effect and to destroy this area to achieve cure. After irradiation, necrosis and fibrosis of the tumor cells can lead to remission of the focus, which might increase the rate of sphincter preservation and R0 resectability for surgery (Glynne-Jones et al. 2018) and improve the local control rate and DFS in the long term (Sebag-Montefiore et al. 2009; Roh et al. 2009). However, there is a trade-off between the benefits of radiotherapy and the risk of side effects (Peeters et al. 2005), including prolonging the operation and causing more blood loss (Enker et al. 1999; Kapiteijn et al. 2001), postoperative pelvic abscess (Enker et al. 1999) and, subsequently, bowel dysfunction (fecal incontinence, blood loss, mucus loss, increased defecation frequency, urinary incontinence and worse social function (Lange et al. 2007; Bruheim et al. 2010; Dahlberg et al. 1998)), along with increased postoperative morbidity (Cedermark et al. 1995; Goldberg et al. 1994). According to a cohort study of stage II/III RC, preoperative radiotherapy fails to improve the retention rate of the anal sphincter) (Bosset et al. 2006). Similarly, in the current study, the anal preservation rates were comparable between the N-CRT and N-CT groups (87.5% of N-CRT versus 94.2% of N-CT). In general, a satisfactory outcome of anal preservation was achieved in this work (90.2%). A higher incidence was observed in men (approximately 70%), which is consistent with reports of the epidemiologic features of CRC (Xie et al. 2021). According to a recent study, local excision could be proposed in selected patients having a small T2/T3 low RC with a good clinical response after chemoradiotherapy (Rullier et al. 2020). Here, the only patient who received transanal resection with a tumor stage from cT3N0 to ypT1N0 had a favorable OS of 70.7 months without recurrence. Sphincter preservation for RC has been taken more and more attention for its role in the improvement of defecation control, sexual function and quality of life after surgical resection. Even so, bowel dysfunction could be observed in 25–90% of patients, with wide-ranging symptoms collectively known as anterior resection syndrome (ARS) or low anterior resection syndrome (LARS) (Bregendahl et al. 2013). It is important to note that most studies report that severe LARS occurs in 40–60% of RC patients after anus-preserving operations (Bregendahl et al. 2013; Ziv et al. 2013; Emmertsen and Laurberg 2013). A substantial subset of severe LARS persists, which has a serious impact on patients' quality of life (Emmertsen and Laurberg 2013; Chen et al. 2015). Preoperative radiotherapy was associated with more than twice the risk of severe LARS after radical surgery (Bregendahl et al. 2013; Emmertsen and Laurberg 2013; Chen et al. 2015; Qin et al. 2017). A recent cross-sectional study from China showed that preoperative long-course radiotherapy and a lower third tumor were independently associated with severe bowel dysfunction after low anterior resection (Qin et al. 2017).

Preoperative radiotherapy does not increase the long-term OS rate of all populations according to most studies (Enker et al. 1999; Kapiteijn et al. 2001; Goldberg et al. 1994). Total neoadjuvant therapy, defined as multi-agent chemotherapy given at least 2 months before RT followed by preoperative chemoradiation therapy and definitive surgery without adjuvant chemotherapy, failed to improve OS and circumferential resection margin as reported previously (Zhu et al. 2019; Goffredo et al. 2021). The results of our study also support this conclusion both internally (*P* = 0.737) and externally (adjusted *P* = 0.089). More importantly, this conclusion was confirmed in TNM stage II and III subgroups in both our center and the SEER database. In the present study, the 5-year OS rate was 89.0% for the N-CRT group versus 87.6% for the N-CT group, and the 5-year DFS rate was 72.5% for the N-CRT group versus 71.6% for the N-CT group, which seems slightly higher than those in reported previous studies (Enker et al. 1999; Kapiteijn et al. 2001; Bosset et al. 2006; Rödel et al. 2015). In addition, radiotherapy is not guaranteed to improve the overall rate of recurrence (local recurrence plus distant recurrence) (Kapiteijn et al. 2001). In our study, the overall 5-year rate of recurrence was 28.1%, but the local recurrence rate was only 4.1% after RC resection, regardless of whether the patients received preoperative radiotherapy. Obviously, disease progression is mainly due to distant metastasis rather than local recurrence, and preoperative local radiotherapy of LARC might be ineffective in this situation. This study was conducted in a high-volume surgery center, and all operative procedures were performed by experienced surgeons to assure the quality of surgical operation, which might partially explain the low local recurrence rate in our study.

The effect of irradiation on anastomotic complications after LARC resection has been a major concern. The incidence of clinical AF (grade B + grade C) after resection varied from 3 to 12% in prospective research (Sauer et al. 2004; Sebag-Montefiore et al. 2009; Fleshman et al. 2015; Qin et al. 2016). Ileostomy could relieve symptoms, especially those accompanied by peritonitis, but failed to prevent AF. The association between radiation and AF is still controversial. Some studies did not find a direct association with preoperative short-course radiotherapy and postoperative AF (Marijnen et al. 2002; Sebag-Montefiore et al. 2009), and a meta-analysis observed no correlation between preoperative radiotherapy and post-surgical AF (Qin et al. 2014). However, other research offered a countervailing point of view. It revealed that preoperative radiotherapy independently increased the risk of post-surgical AF (Qin et al. 2016; Eriksen et al. 2005; Jestin et al. 2008), and clinical AF could lead to the development of stenosis (Qin et al. 2016). Subsequently, anastomotic stenosis could cause dysfunction in bowel evacuation and even obstruction (Luchtefeld et al. 1989). In addition, postoperative AF was reported to be associated with increased morbidity and mortality, as well as potentially unsatisfactory clinical outcomes (Mirnezami et al. 2011; Espín et al. 2015). Apparently, AF can deteriorate quality of life, increase hospitalization expenses and time, and potentially affect oncological outcomes. Thus, it is essential to prevent AF. The incidence of clinical AF in our center was 10.6% (22/208), which is comparable to the results of other reported studies (Sauer et al. 2004; Sebag-Montefiore et al. 2009; Fleshman et al. 2015; Qin et al. 2016).

Most large prospective trials have revealed that the addition of oxaliplatin to fluorouracil-based N-CT fails to improve tumor response or survival (including OS and DFS) (Gérard et al. 2010; Aschele et al. 2011; O'Connell et al. 2014), which is why patients in the N-CR group were not distinguished by the addition or absence of oxaliplatin in this research. Notably, postoperative chemoradiotherapy was not included in the analysis. Adjuvant chemotherapy was implemented at the physician’s discretion and with agreement from the patient. In the current study, the proportion of adjuvant chemotherapy between the N-CRT and N-CT groups was roughly comparable (98.1% versus 92.3%, *P* = 0.052). Postoperative radiotherapy is not implemented routinely and was thus not included in the study. In addition, ileostomy has not yet been taken into account because it is not regarded as protective against AF. The pCR rate after neoadjuvant therapy in the literature varies from 13.9 to 19.2%. Specifically, it was 17.8% in NSABP R-04, 16.0% in STAR-01, 13.9% without oxaliplatin and 19.2% with oxaliplatin in ACCORD 12/0405-Prodige 2. A total of 33 (15.9%) patients who received N-CRT or N-CT achieved pCR in this study, which is analogous to previous research (Gérard et al. 2010; Aschele et al. 2011; O'Connell et al. 2014). We carried out an up to 5-year long-term follow-up, which is longer than that of the FOWARC trial (median of 45.2 months) (Deng et al. 2019).

There are some limitations to this study. First, this is a retrospective single-center study with inherent defects. Second, although our hospital is a high-volume center for CRC, the total number of participants is still limited. In addition, shortcomings from the retrospective nature of this study are unavoidable; hence, multi-center, prospective research can be a feasible direction in the future. Data on recurrent events are unavailable in the 18 population-based SEER registries. The conclusions presented are based on long-course radiotherapy, and whether the results of our study can be applied to patients with short-course radiotherapy is unknown.

In summary, for the management of LARC, N-CT alone can achieve comparable survival benefits as N-CRT and has a lower incidence of complications than N-CRT. Therefore, physicians should delicately judge and weigh the benefits and negative effects of radiotherapy before N-RT. Multimodal therapy involving surgery, chemoradiation and other individualized treatments is essential, and the administration of radiotherapy should be finely balanced to achieve the maximum possible benefit in survival and quality of life in the future.

## Supplementary Information

Below is the link to the electronic supplementary material.

Supplementary file1 (PDF 929 KB)

Supplementary file2 (XLSX 12 KB)

Supplementary file3 (XLSX 11 KB)

Supplementary file4 (XLSX 9 KB)

Supplementary file5 (XLSX 10 KB)

Supplementary file6 (XLSX 10 KB)

Supplementary file7 (XLSX 9 KB)

## Data Availability

The datasets generated during and/or analyzed during the current study are available from the corresponding author on reasonable request.
