# Supplementary material for: Neoadjuvant chemoradiotherapy versus neoadjuvant chemotherapy alone for patients with locally advanced rectal cancer: a propensity-score-matched analysis combined with SEER validation
Source: J Cancer Res Clin Oncol. 2023 May 8;149(11):8897–912. doi: 10.1007/s00432-023-04779-y (PMC10374480; doi:10.1007/s00432-023-04779-y)
Supplement: Supplementary file 1 — Supplementary file1 (PDF 929 KB) [file 432_2023_4779_MOESM1_ESM.pdf]

Supplementary Figure 1: Flow chart illustrating of patients selection in SEER database.

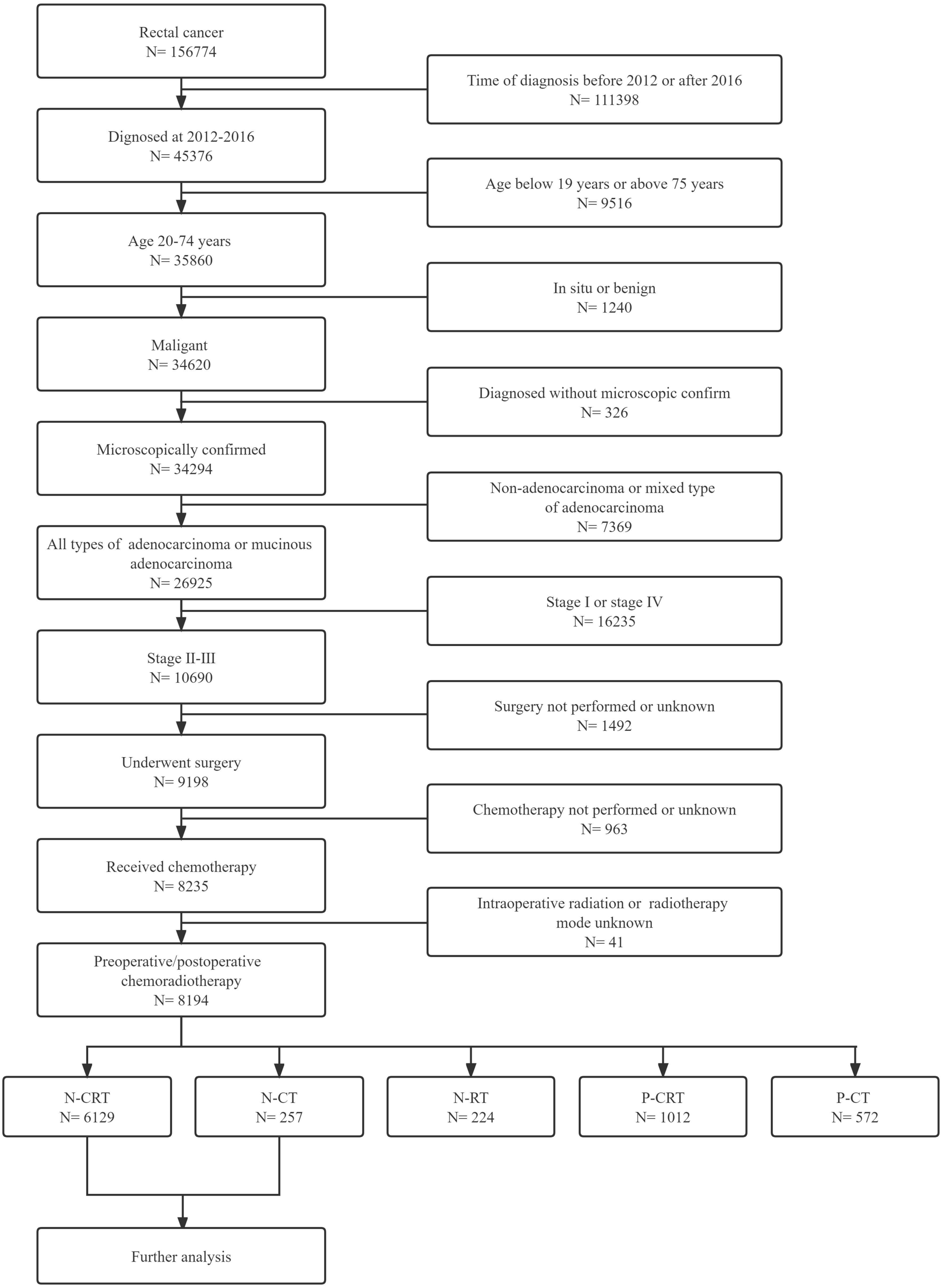

Supplementary Figure 2: Survival analysis of stage II LARC between the N-CRT and N-CT group for overall survival (a), disease-free survival (b), cancer-specific survival (c) and locoregional recurrence-free survival (d) at our center.

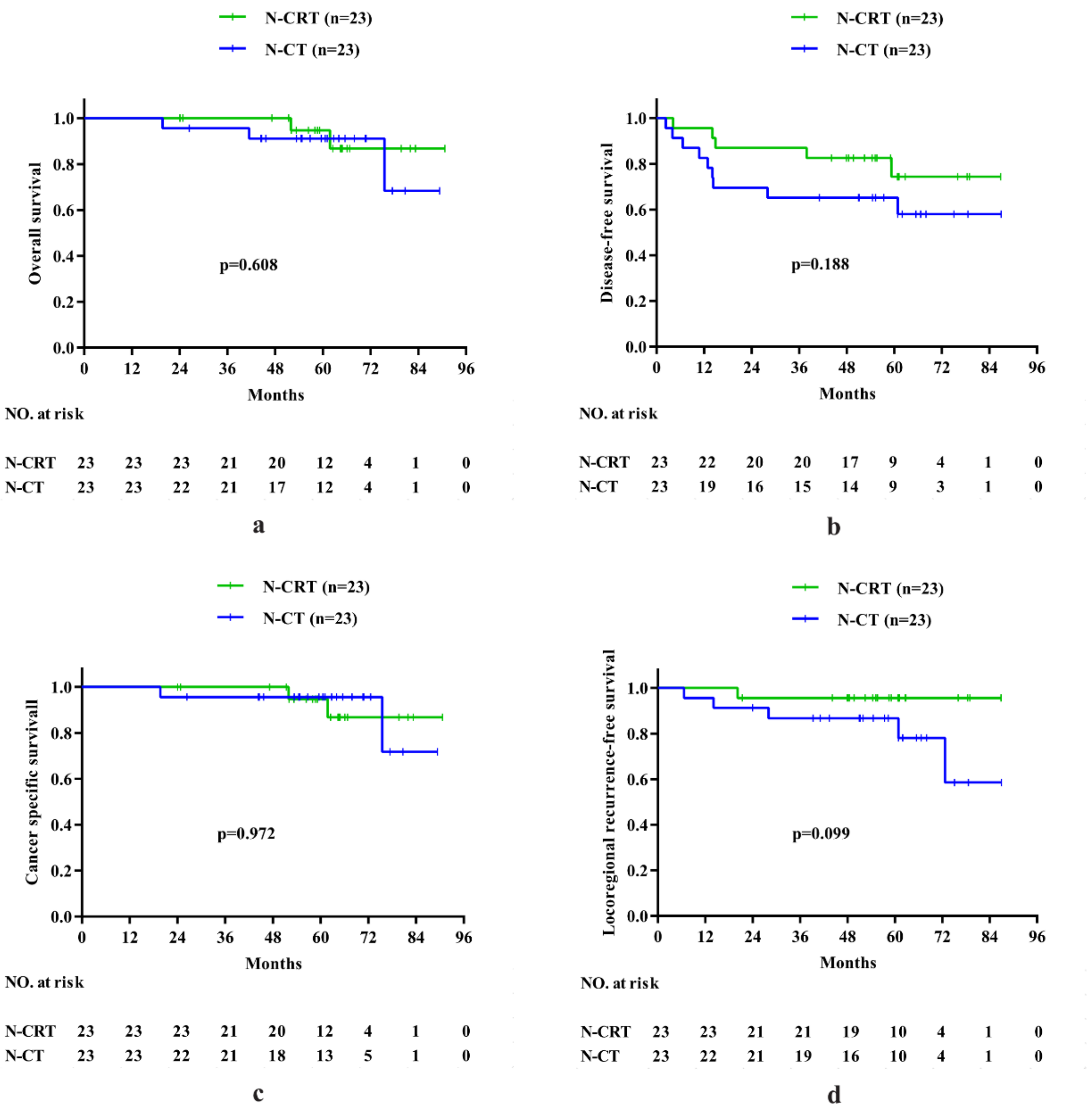

Supplementary Figure 3: Survival analysis of stage III LARC between the N-CRT and N-CT group for overall survival (a), disease-free survival (b), cancer-specific survival (c) and locoregional recurrence-free survival (d) at our center.

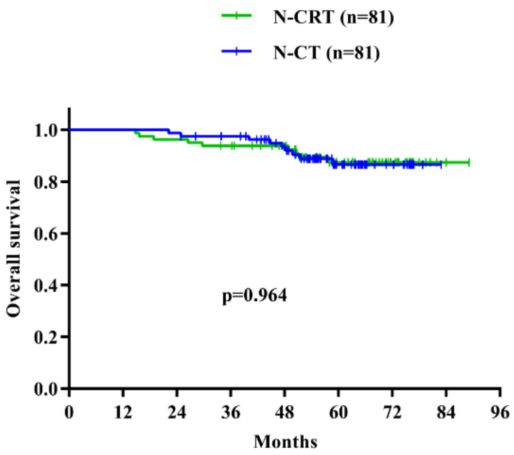

NO. at risk

	0	12	24	36	48	60	72	84	96
N-CRT	81	81	78	75	66	39	20	2	0
N-CT	81	81	80	76	65	34	12	0	0

a

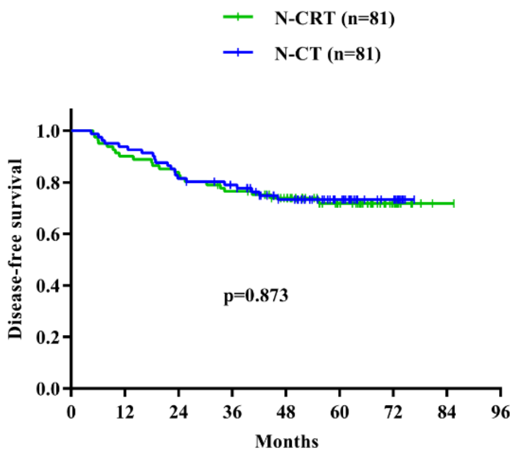

NO. at risk

	0	12	24	36	48	60	72	84	96
N-CRT	81	73	68	60	49	31	13	1	0
N-CT	81	76	66	61	46	23	11	0	0

b

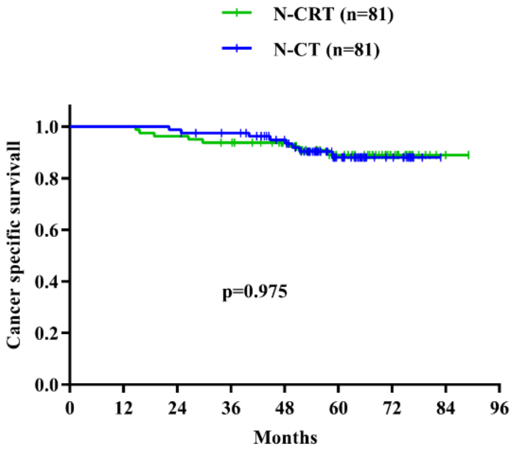

NO. at risk

	0	12	24	36	48	60	72	84	96
N-CRT	81	81	78	75	66	40	20	2	0
N-CT	81	81	80	76	66	35	12	0	0

c

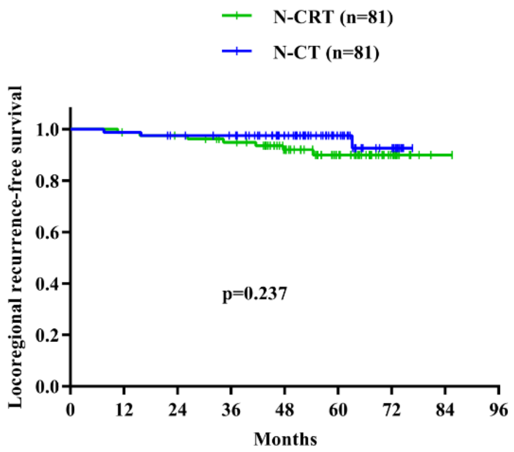

NO. at risk

	0	12	24	36	48	60	72	84	96
N-CRT	81	79	77	72	57	35	13	1	0
N-CT	81	80	77	74	59	27	11	0	0

d

Supplementary Figure 4: Survival analysis of all LARC patients between the N-CRT and N-CT group for overall survival in SEER database.

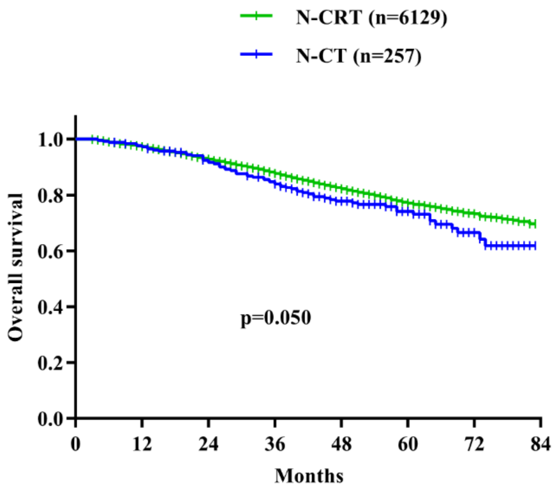

NO. at risk

N-CRT	6129	5934	5597	5213	3632	2124	967	0
N-CT	257	249	228	206	138	80	32	0
